# Sinefungin, a Natural Nucleoside Analogue of S-Adenosylmethionine, Inhibits *Streptococcus pneumoniae* Biofilm Growth

**DOI:** 10.1155/2014/156987

**Published:** 2014-06-23

**Authors:** Mukesh Kumar Yadav, Seok-Won Park, Sung-Won Chae, Jae-Jun Song

**Affiliations:** ^1^Department of Otorhinolaryngology-Head and Neck Surgery, Korea University College of Medicine, Seoul 135-705, Republic of Korea; ^2^Department of Otorhinolaryngology-Head and Neck Surgery, Dongguk University Ilsan Hospital, Gyeonggi, Goyang 410-773, Republic of Korea

## Abstract

Pneumococcal colonization and disease is often associated with biofilm formation, in which the bacteria exhibit elevated resistance both to antibiotics and to host defense systems, often resulting in infections that are persistent and difficult to treat. We evaluated the effect of sinefungin, a nucleoside analogue of S-adenosylmethionine, on pneumococcal *in vitro* biofilm formation and *in vivo* colonization. Sinefungin is bacteriostatic to pneumococci and significantly decreased biofilm growth and inhibited proliferation and structure of actively growing biofilms but did not alter growth or the matrix structure of established biofilms. Sinefungin significantly reduced pneumococcal colonization in rat middle ear. The quorum sensing molecule (autoinducer-2) production was significantly reduced by 92% in sinefungin treated samples. The *luxS, pfs*, and *speE* genes were downregulated in biofilms grown in the presence of sinefungin. This study shows that sinefungin inhibits pneumococcal biofilm growth *in vitro* and colonization *in vivo*, decreases AI-2 production, and downregulates *luxS*, *pfs*, and *speE* gene expressions. Therefore, the S-adenosylmethionine (SAM) inhibitors could be used as lead compounds for the development of novel antibiofilm agents against pneumococci.

## 1. Introduction

*Streptococcus pneumoniae *is a major cause of pneumonia, meningitis, bacteremia, and otitis media (OM) in young children and the elderly [1, 2]. Pneumococcal colonization and disease is often associated with biofilm formation [3], in which the adherent bacteria are attached to the host surface and to each other to form multilayer structures covered by an extracellular matrix (exopolysaccharide (EPS)) [4, 5]. Bacteria in biofilms exhibit elevated resistance both to antibiotics and to host defense systems, which often results in infections that are persistent and difficult to treat [6, 7]. Various studies have previously reported that pneumococcal biofilm exhibits significantly higher resistance to penicillin, tetracycline, rifampicin, amoxicillin, erythromycin, clindamycin, and levofloxacin [8, 9]. Marks et al. reported that pneumococcal biofilms formed on nasopharyngeal tissue of mouse were more resistant to gentamicin and penicillin G than did planktonic cell [10]. Therefore, there is an urgent need to search for alternative antimicrobial target to develop antimicrobial compound against pneumococcal biofilms.

The effective antibiofilm strategies should include inhibition of microbial adhesion to the surface and of colonization and interference with the signal molecules modulating biofilm development and the disaggregation of the biofilm matrix [11, 12]. In* S. pneumoniae*, quorum sensing (QS) signaling regulates biofilm communities and plays a key role in coordinating the spatial disposition, aggregation of cells, and exopolysaccharide formation [13, 14]. Autoinducer-2 (AI-2) is the only QS molecule in pneumococci which is synthesized by S-ribosylhomocysteine lyase (LuxS) through activated methyl cycle (AMC) [15]. In AMC, the S-adenosyl-L-methionine (SAM) is a central molecule which donates methyl group for methionine recycling, methylation of biomolecules, and biosynthesis of AI-2 [16, 17]. Furthermore, the byproducts of AMC such as 5′-methylthioadenosine/S-adenosylhomocysteine (MTA/SAH) are toxic to the bacteria [15]. In* S*.* pneumoniae* detoxification of SAH is carried out by MTA/SAH nucleosidase (Pfs) and S-ribosylhomocysteine lyase (LuxS), and this pathway is absent in human [17–19].

The SAM is a central molecule of AMC involved in recycling of methionine, methylation of biomolecules, biosynthesis of AI-2, and polyamine biosynthesis [20, 21]. Therefore, we hypothesized that altering the SAM activity could have adverse effect on pneumococcal biofilms. Here we evaluated the effect of sinefungin, a nucleoside analogue of S-adenosylmethionine, on pneumococcal* in vitro* biofilm.

Sinefungin is a natural nucleoside and a structural analog of SAM. It inhibits transmethylation reactions related to DNA, RNA, proteins, and other molecules [22–25]. Antifungal, antiviral, and antiprotozoal activities of sinefungin have been reported [26, 27]. Previously, Parsek et al. studied effect of sinefungin on* Pseudomonas* acyl homoserine-lactone quorum-sensing signal generation [28]. However, the effect of sinefungin on* S. pneumoniae* has not been studied. In this study we examined the effect of sinefungin on* S. pneumoniae*, particularly on biofilm growth.

## 2. Materials and Methods

### 2.1. Bacteria Strains and Culture Conditions

*S. pneumoniae* D-39 strain (NCTC 7466) is an encapsulated, serotype 2 pathogenic strain. It was obtained from the Health Protection Agency Culture Collections (HPA, Salisbury, UK). Bacteria were routinely grown in tryptic soy broth (TSB; BD Difco, Detroit, MI, USA) or on blood agar (BA; Hye In, Seoul, Korea) supplemented with 5% v/v sheep blood at 37°C in an atmosphere of 5% CO_2_.* Vibrio harveyi (V. harveyi*) strains MM32 (ATCC BAA-1121) [29] were grown on autoinducer bioassay (AB) medium at 30°C [30]. Sinefungin (sc-203263) was purchased from Santa Cruz Biotechnology (Santa Cruz, CA, USA).

### 2.2. Effect of Sinefungin on Planktonic Cell Growth

*S. pneumoniae* were grown with different concentrations of sinefungin (10, 20, 30, 40, and 50 *μ*g mL^−1^) at 37°C in 5% CO_2_ for different time points (0 h–10 h). And optical density at 600 nm (OD_600_) was measured after 2 h time interval using a SpectraMax plus 384 automated microplate reader (Molecular Devices, Sunnyvale, CA, USA). After 6 h of incubation, 100 *μ*L aliquots was serially diluted and plated on BA for enumeration of colony forming units (CFU). The percentage decrease in planktonic cell growth was calculated by subtracting the values of sinefungin treated samples from control (untreated) samples. The experiment was replicated three times with triplicate samples at each time point.

### 2.3. Effect of Sinefungin on* In Vitro* Biofilm Growth

*In vitro* pneumococcal biofilm growth was carried out in 96-well, flat-bottom, polystyrene microtiter plate (BD falcon, Sparks, MD, USA) using a static model [31]. Briefly, a fresh colony of* S. pneumoniae* grown overnight on BAP was scraped and grown in TSB+1% glucose medium [32]. The mid-logarithmic phase cell suspension (1 × 10^8^ CFU mL^−1^) was diluted 1 : 100 with fresh sterile TSB+1% medium and 200 *μ*L aliquots were inoculated in wells of 96-well microtiter plates and incubated at 37°C in 5% CO_2_. After incubation, the medium was discarded and plates were gently washed three times with 200 *μ*L sterile phosphate buffered saline (PBS). Thereafter, plates were air-dried and stained with 50 *μ*L crystal violet (CV; 0.1%) for 15 min. Excess stain was decanted off and plates were washed three times with sterile distilled water. The biofilm was dissolved in 200 *μ*L of 95% ethanol and the OD_570_ nm was measured using the aforementioned automated spectrophotometer. The data represent the average value of three replicates.

### 2.4. Effect of Different Concentrations of Sinefungin on Biofilm Growth

To study the concentration-dependent effect of sinefungin on pneumococcal biofilms,* S. pneumoniae* biofilms were grown with 10–50 *μ*g mL^−1^ sinefungin for 18 h as described above. Biofilm biomass was detected by CV-microtiter plate assay as described above. The percentage decrease in biomass was calculated by subtracting the biomass of control biofilms grown without sinefungin. Enumeration of biofilm bacteria was done as described above (i.e., CFU).

### 2.5. Effect of Sinefungin on Established Biofilms

Pneumococcal biofilms were established in microtiter plate wells for 12 h. The formed biofilms were exposed to different concentrations of sinefungin for 6 h and analyzed by CV-microtiter plate assay and the cell viability was determined by CFU counts.

### 2.6. Effect of Different Exposure Times of Sinefungin on Biofilm Formation

Pneumococcal biofilms were generated in the presence of 35 *μ*g mL^−1^ sinefungin for 6, 12, 18, and 24 h. At each time point, biofilm biomass was determined as described above.

### 2.7. Scanning Electron Microscopy (SEM) Biofilms

*In vitro* biofilms grown with 35 *μ*g mL^−1^ sinefungin and without sinefungin in 24-well tissue culture plates for 18 h were analyzed by SEM. The medium was removed and the plates were gently washed two times with sterile PBS to remove planktonic cells. The samples were prefixed by immersion in 2% glutaraldehyde in 0.1 M phosphate buffer and postfixed for 2 h in 1% osmic acid dissolved in PBS. Samples were treated in a graded series of ethanol and t-butyl alcohol, dried in a model ES-2030 freeze dryer (Hitachi, Tokyo, Japan), platinum coated using an IB-5 ion coater (Eiko, Kanagawa, Japan), and observed using a S-4700 field emission scanning electron microscopic (Hitachi).

### 2.8. Autoinducer Assay

*V. harveyi *MM32 was used as the qualitative reporter strain to detect the changes in the production of AI-2.* V. harveyi* MM32 (BB120 luxN::Tn5 luxS::Tn5) is an ideal reporter strain as it can sense AI-2 but cannot synthesize AI-2 of its own [29]. The reporter strain was grown in AB medium for 16 h and then diluted 1 : 5,000. A total of 90% of the diluted reporter strain was then added to a 96-well plate.* S. pneumoniae* biofilms were grown with 35 *μ*g mL^−1^ sinefungin and without sinefungin as described earlier for 18 h and sonicated for 3 seconds to disperse the adherent cells. Then 1 mL of the cell suspension was centrifuged and filter sterilized. And 10% of the filtered supernatant was added to the 90% reporter stain. Media without bacteria were negative control. The plates were incubated at 30°C for 15 h and the luminescence of the reporter strain was monitored using a luminometer (GloMax Multi-Detection System Promega, Madison, WI, USA).

### 2.9. *In Vivo* Pneumococcal Colonization

For* in vivo* colonization study, we used rat OM model. The animal experiment protocol was reviewed and approved by the animal research and care committee at Dongguk University Ilsan Hospital (Gyeonggi, South Korea). Twenty pathogen-free, Sprague Dawley rats weighing 150–200 g were obtained from Orient Bio (Gyeonggi, South Korea). All rats were examined prior to use by otomicroscopy to document abnormal middle ear and were kept isolated in an infection-free zone for 2 weeks. Rats were assigned randomly to groups that received bacteria (*n* = 8), bacteria with 35 *μ*g mL^−1^ sinefungin (*n* = 8), or no procedure (control group, *n* = 4). Fifty microliters of cell suspension containing 3 × 10^7^ CFU* S. pneumoniae* was injected into the middle ear cavity through the tympanic membrane of the right ear using a tuberculin syringe and a 27-gauge needle [33]. Animals were sacrificed 1 week after inoculation and the middle ear bulla was aseptically acquired. The tympanic membrane was removed and ears were irrigated to remove planktonic bacteria. The bullae were homogenized and serially diluted and plated on BAP for enumeration of CFU.

### 2.10. Quantification of Gene Expression of* In Vitro* Formed Biofilms Using Quantitative Real-Time RT-PCR

Expressions of* luxS*,* pfs,* and* speE* genes in biofilms grown for 18 h without and with (35 *μ*g mL^−1^) sinefungin were quantified by real-time RT-PCR. The biofilms were washed and the adherent cells were scraped and immediately processed for RNA extraction. The cells were lysed by incubation of the cell pellet in 100 *μ*L (3 mg mL^−1^) lysozyme (Sigma-Aldrich, St. Louis, MO, USA) for 4 min. The total RNA was extracted using a RNeasy Total RNA Isolation System Kit (Qiagen, Valencia, CA, USA) according to the manufacturer's instructions with few modifications. On-column DNAse (Qiagen) treatment was performed for 10 min at 20–25°C. RNA quality was assessed spectrophotometrically.

cDNA was synthesized using the ImProm-II Reverse Transcriptase Kit (Promega, Madison, WI, USA) according to the manufacturer's instructions. Primers for* speE* gene were designed by standard procedures from the nucleotide sequence of* S. pneumoniae* D39 strain (Table 1). The primers used for* luxS*,* pfs, *and* gyrB* (house-keeping) genes were previously reported [31, 34]. Real-time RT-PCR was carried out in total volume of 20 *μ*L, consisting of 10 *μ*L of 2X SYBR Green PCR Master Mix (Roche Applied Science, Indianapolis, IN, USA), 3 pmol of each forward and reverse primers, 4 *μ*L cDNA, and nuclease-free water. PCR conditions included initial denaturation at 95°C for 10 min, followed by 45 cycles of denaturation at 95°C for 15 sec, annealing at 56°C for 10 sec, extension at 72°C for 15 sec, and final extension at 72°C for 5 min, followed by melting curve analysis from 60 to 95°C. Negative controls containing nuclease-free water and no reverse transcriptase control were included in each RT-PCR experiment. The relative gene expression was analyzed using the 2^−ΔΔCT^ method [35]. The reference gene was* gyrB *and the standard condition was biofilms grown without sinefungin.

### 2.11. Statistical Analysis

The values were calculated as the mean of individual experiments performed in triplicate and compared with those of the control groups. Differences between two mean values were calculated by Student's *t*-test. The statistically significant tests were set at a *P* value <0.05.

## 3. Results

### 3.1. Effect of Sinefungin on Planktonic Cell Growth

**Figure 1:**
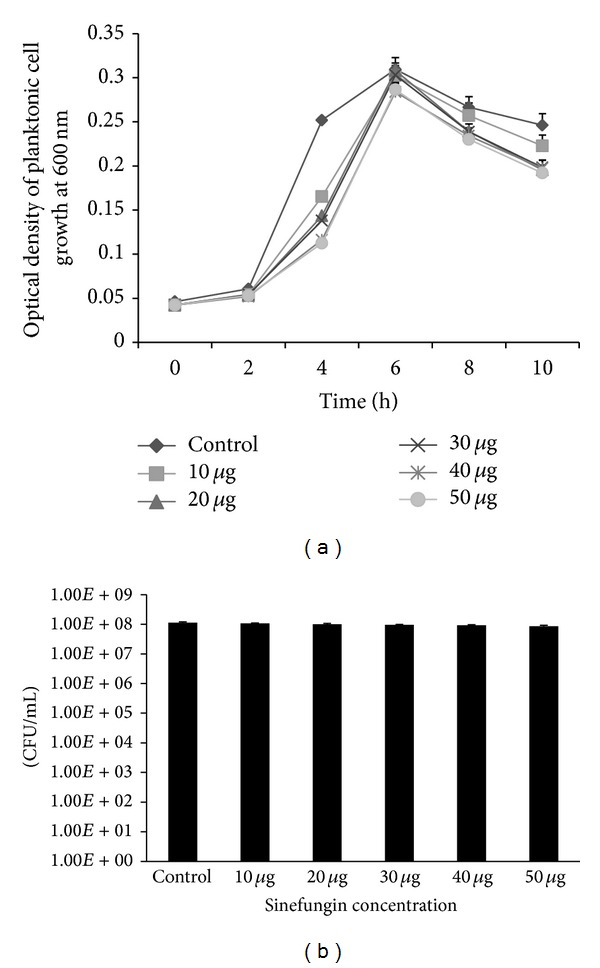
(a) Growth of* Streptococcus pneumoniae* with different concentrations of sinefungin (10 *μ*g to 50 *μ*g mL^−1^) in time course experiment. 1 : 100 diluted cell suspensions were grown with different concentrations of sinefungin at different time points (0, 2, 4, 6, 8, and 10 h) at 37°C in 5% CO_2 _and optical density was measured at 600 nm. (b) CFU counts of pneumococci grown with different concentrations of sinefungin at 6 hours. After 6 h of incubation, 100 *μ*L aliquots were serially diluted and plated on blood agar plate to enumeration bacteria. Error bars represent the standard deviation of the mean values.

The growth of pneumococci with different concentrations of sinefungin in time course experiment showed bacteriostatic effect of sinefungin. The growth of bacteria in sinefungin treated samples was slow in comparison to control samples (untreated). And a significant maximum growth difference in sinefungin treated and control samples was detected at mid-log phase (4 h after inoculation). However, at the end of the log phase (6 h of inoculation) there was no significant planktonic bacteria growth inhibition (Figure 1(a)). The CFU count of 6 h grown bacteria also detected no significant difference in sinefungin treated and untreated samples (Figure 1(b)).

### 3.2. Effect of Sinefungin on* In Vitro* Biofilm Growth

#### 3.2.1. Sinefungin Inhibits* In Vitro* Formation of Pneumococcal Biofilms

**Figure 2:**
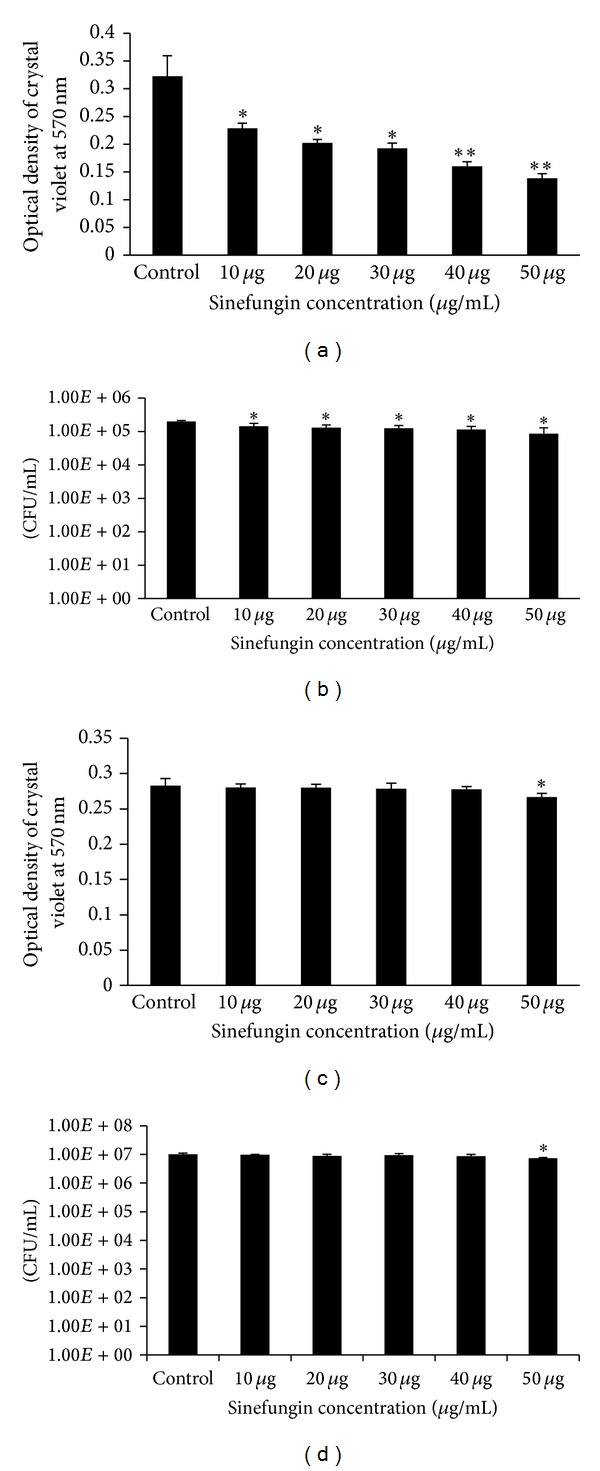
Effect of sinefungin on pneumococcal* in vitro* biofilm growth. (a), (b) Pneumococcal biofilm growth with different concentrations of sinefungin for 18 h and detection of biofilm biomass by crystal-violet microtiter plate assay and CFU counts of pneumococcal biofilms. (c) and (d) show the effect of different concentrations of sinefungin on already established biofilms. (c) Detection of biofilm biomasses with crystal-violet microtiter assay. (d) CFU counts of pneumococcal biofilms. The error bars represent the standard deviation of the mean values. Significance was determined by Student's *t*-test (*P < 0.05, **P < 0.01).

Significantly (*P* < 0.05) decreased biofilm biomass and CFU counts were detected in the samples grown with sinefungin. The inhibitory effect of sinefungin was concentration-dependent (Figures 2(a) and 2(b)). Biofilms grown with 10 *μ*g mL^−1^ (lowest concentration) and 50 *μ*g mL^−1^ (highest concentration) sinefungin demonstrated a significant (*P* < 0.05) biomass decrease of 15% and 53%, respectively.

#### 3.2.2. Effect of Sinefungin on Established Biofilms

Pneumococcal biofilms were generated for 12 h and then incubated with different concentrations of sinefungin for 6 h. No significant decrease of biofilm biomass and viable count was evident at 10, 20, 30, and 40 *μ*g mL^−1^ of sinefungin, while 50 *μ*g mL^−1^ sinefungin produced significant decrease in biofilm biomass and viable counts, although the percentage decrease was low (only 5% of biofilm decreases) (Figures 2(c) and 2(d)).

#### 3.2.3. Biofilm Inhibition with Time

**Figure 3:**
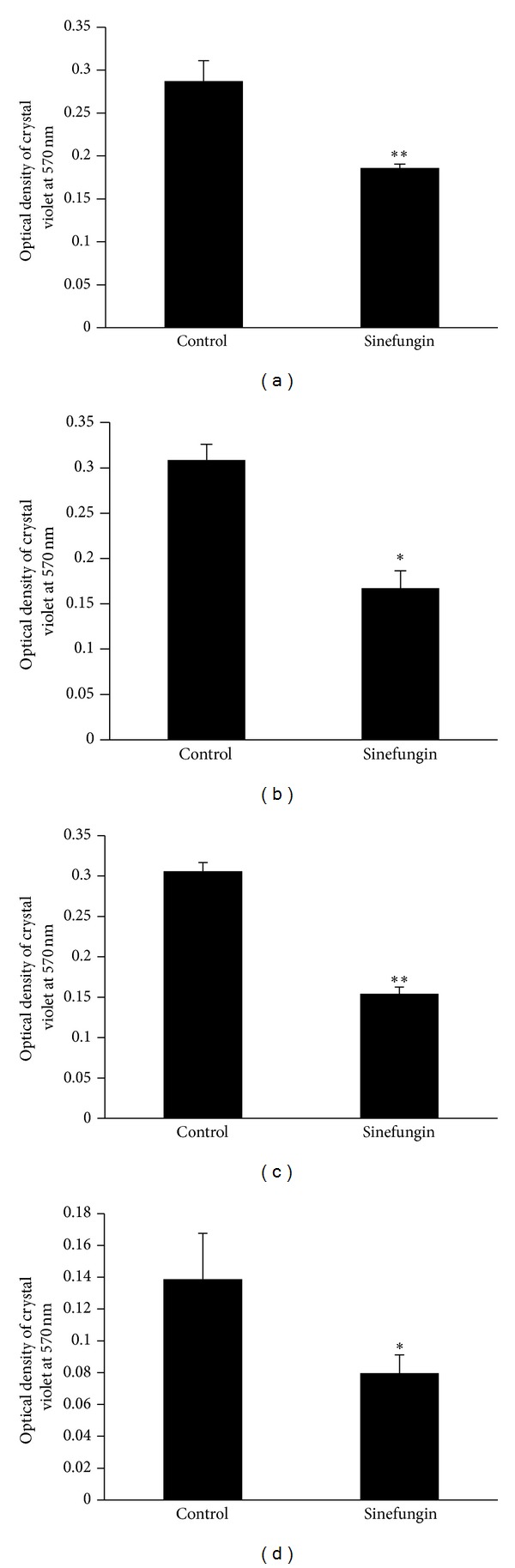
Inhibitory effect of sinefungin (35 *μ*g mL^−1^) on* Streptococcus pneumoniae in vitro* biofilms grown at different time intervals (6, 12, 18, and 24 h). Pneumococcal biofilms were grown with 35 *μ*g mL^−1^ sinefungin at different time points and the biofilm biomasses were quantified by crystal-violet microplate assay. (a) Biofilms grown at 6 h; (b) biofilms grown at 12 h; (c) biofilms grown at 18 h; and (d) biofilms grown at 24 h. The experiments were repeated three times in triplicate. The error bars represent the standard deviation of the mean values. The results were significant by Student's *t*-test (*P < 0.05, **P < 0.005).

To determine the inhibitory effect of sinefungin on biofilms grown at different times, biofilms were generated for 6, 12, 18, and 24 h with and without sinefungin. A significant (*P* < 0.05) decrease of biofilm biomass was evident at all time points (Figure 3).

### 3.3. SEM of Pneumococcal Biofilms

**Figure 4:**
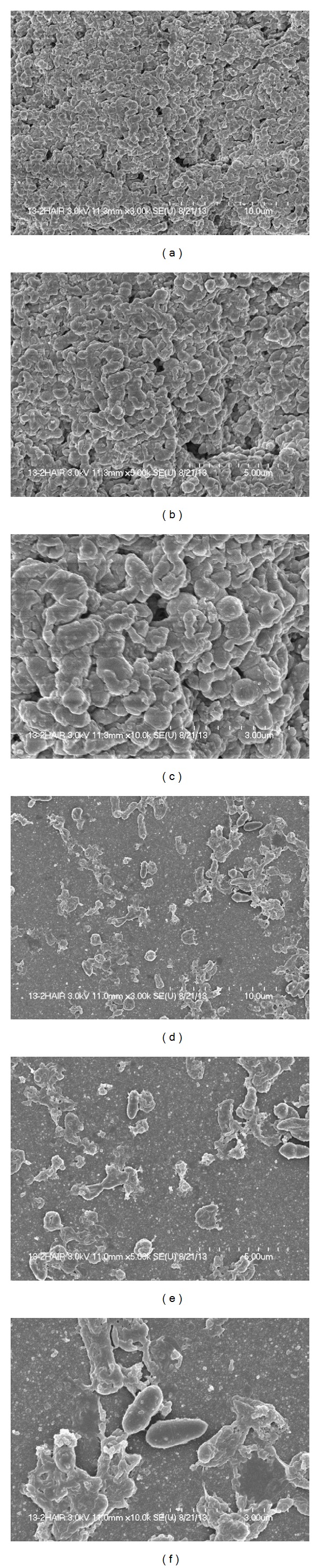
Scanning electron microscope (SEM) images of biofilms grown with and without sinefungin. Panels (a), (b), and (c) are representative of SEM images of biofilms without sinefungin; the biofilm is thick and well organized with a three-dimensional structure. Panels (d), (e), and (f) are representative of SEM images of biofilm grown with 35 *μ*g mL^−1^ sinefungin; biofilms are thin and scattered; and clumps of cells are seen with no organized structure. The scale bars of the figure are 10, 5, and 3 *μ*m.

Biofilms grown in the absence and presence (35 *μ*g mL^−1^) of sinefungin were examined by SEM. Highly organized biofilm was produced in the absence of sinefungin. These biofilms were heterogeneous in nature, with cells embedded in the matrix and connected with each other and to the base of the plate. The adherent cells formed a three-dimensional structure of significant depth (Figures 4(a), 4(b), and 4(c)). In contrast, biofilms grown with 35 *μ*g mL^−1^ sinefungin were thin and the bacteria were scattered in clumps. Cell-to-cell connections were not visible and the biofilms were disorganized (Figures 4(d), 4(e), and 4(f)).

### 3.4. Autoinducer Assay

**Figure 5:**
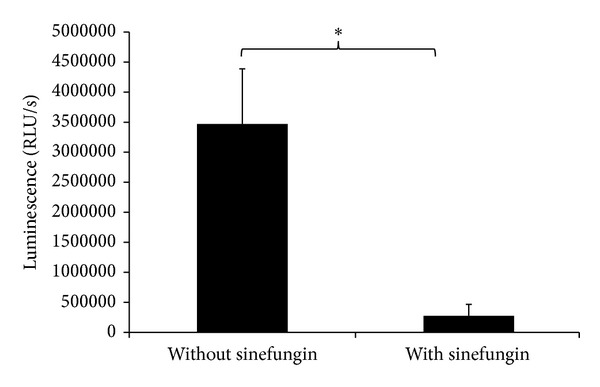
Measurement of autoinducer-2 production changes in cell free supernatant of biofilms grown with 35 *μ*g mL^−1^ sinefungin and without sinefungin using* vibrio harveyi* reported strain.* V. harveyi* reporter strain was grown with cell free supernatant for 15 h and luminescence response was measured. The error bars represent the standard deviation of the mean values. Significance was determined by Student's *t*-test (*P < 0.05).

The cell-free supernatants of biofilms grown with and without sinefungin were examined for AI-2 production via the* V. harveyi *MM32 reporter strain (Figure 5). After 15 h, the reporter strain detected significantly high AI-2-induced luminescence response in the supernatant from the untreated samples than samples grown with sinefungin. The luminescence responses were significantly 92% less in sinefungin treated samples in comparison to untreated samples. These results indicate that sinefungin interferes and decreases the production of AI-2 in pneumococcal biofilms.

### 3.5. Sinefungin Inhibits* In Vivo* Pneumococcal Colonization

**Figure 6:**
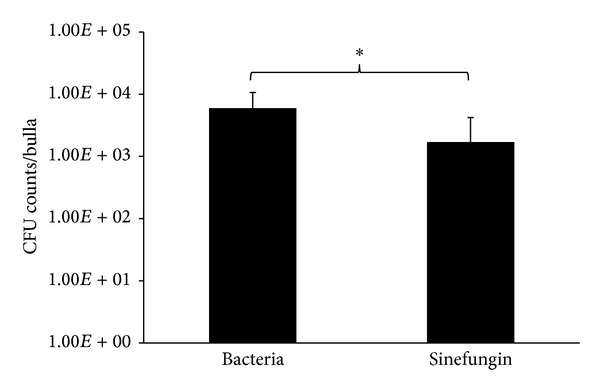
Quantification of pneumococcal colonization in the middle ear of rats inoculated with 35 *μ*g mL^−1^ sinefungin. CFU count was used to enumerate bacteria in whole bulla lysate of samples treated with bacteria suspension supplied with sinefungin with respect to control groups. The error bars represent the standard deviation of the mean values. The results were significant by Student's *t*-test (*P < 0.05).

The* in vivo* experiment showed less bacterial recovery in bulla lysate isolated from rat inoculated with sinefungin after 1 week of bacterial inoculation. The mean CFU counts of bacteria only treated group were 5.98 × 10^3^ (*SD* = 4649) and of sinefungin treated group were 1.70 × 10^3^ (*SD* = 2533). A significant (*P* < 0.05) 70% less bacteria were recovered in sinefungin treated rat bulla (Figure 6).

### 3.6. Quantification of Gene Expression of* In Vitro* Biofilms

**Figure 7:**
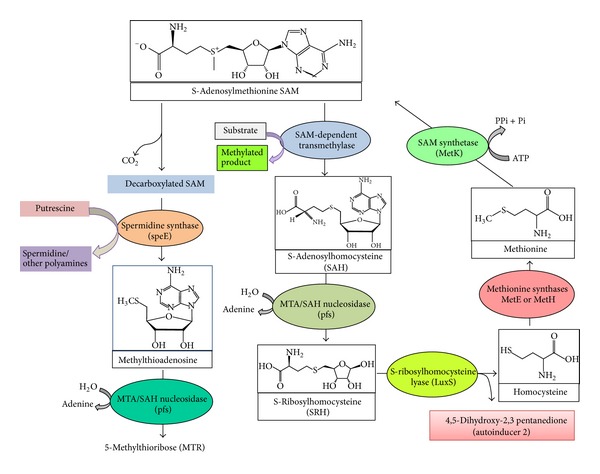
Systematic diagram of cyclic production of homocysteine and activated methyl cycle in* Streptococcus pneumoniae*. Methionine is converted into SAM by SAM synthetase (MetK). Donation of the methyl group of SAM to a variety of methyl acceptors results in SAH; the MTA/SAH nucleosidase (pfs) first converts SAH into S-ribosylhomocysteine (SRH) which is then recycled back to homocysteine by LuxS. As a byproduct of this reaction, 4,5-dihydroxy 2,3-pentanedione is produced which spontaneously forms autoinducer-2. The decarboxylation of SAM produces MTA (methylthioadenosine) as catalyzed by spermidine synthase (speE). Spermidine (a polyamine) is the byproduct of this reaction from putrescine.

Quantitative RT-PCR revealed a significant (>2-fold) decreased gene expressions of* luxS*,* pfs*, and* speE* in biofilms grown with sinefungin compared to biofilm growth without sinefungin. The expression of* luxS* was significantly decreased by 8.53-fold (*P* = 0.001) in the biofilms grown with sinefungin. Expression of the* pfs *gene was decreased by 2.88-fold (*P* = 0.006). Similarly, the* speE* gene was downregulated by 3.22-fold (*P* = 0.01). Gene annotation revealed the identity of the* luxS* and* pfs* genes as methionine pathways genes that recycle cysteine through AMC and the QS molecule AI-2 is the byproduct of AMC (Figure 7). In this pathway, SAM acts as a methyl donor. The* luxS* and* pfs* genes encode enzymes that catalyze removal of SAH and SRH, which are toxic to bacteria. The* speE* gene is involved in biosynthesis of spermidine catalysis by decarboxylated SAM.

## 4. Discussion

SAM is an important nucleoside that serves as an activated methyl group donor in the methylation of macromolecules to yield SAH/MTA which leads to biosynthesis of AI-2 and polyamine. Therefore, inhibition of SAM activity by certain substrate analogs could decrease* luxS* and* pfs* genes expression and limits synthesis of autoinducer-2, and, hence, causes reduction in biofilm formation and may attenuate virulence.

In this study, we examined the effect of sinefungin, a nucleoside analogue of SAM and a methytransferase inhibitor, on pneumococcal biofilm growth* in vitro* and* in vivo* colonization.

Planktonic growth of pneumococci with different concentrations of sinefungin demonstrated that sinefungin is bacteriostatic. The significant inhibitory effect of sinefungin was on actively growing cells (log phase). No significant planktonic cell growth inhibition was detected at concentrations that inhibited biofilm growth, which indicates that sinefungin may be a biofilm growth inhibitor. The inhibitory effect of sinefungin on* in vitro* biofilms was concentration-dependent. We tested the inhibitory effect of sinefungin with time on* in vitro* biofilms grown [36]. At all the time points tested, there were low biofilm biomasses and CFU counts in the biofilm samples grown with sinefungin. Probably, sinefungin inhibits quorum sensing systems (luxS-AI-2) that has been reported to control pneumococcal biofilm formation [14, 37]. No significant decrease in the biofilm biomass and the CFU counts, in established biofilms treated with sinefungin, indicates that sinefungin is unable to kill bacteria. The inhibitory effect of sinefungin has been reported in virus, fungi, and protozoa [26, 38].

The difference in the architecture of the biofilms grown with and without sinefungin was verified with SEM. SEM confirmed that the biofilms grown with sinefungin were significantly different in terms of their connection to substrate and thickness and were unorganised. They also lost their cell-to-cell connections and were devoid of microcolonies, similar to a prior report [14].

Using the rat OM model, we detected significantly low recovery of pneumococci in rat bulla treated with sinefungin. This indicates that sinefungin inhibits* in vivo* colonization of pneumococci. It is previously reported that mutant bacterial strains defective in QS create less potent infections [39]. QS-deficient intranasal* S. pneumoniae* infections in mouse were less effective at spreading to the lungs or the bloodstream [40].

The QS regulates pneumococcal biofilms and AI-2 in the only QS molecule in* S. pneumoniae*. Various studies reported the prevention and treatment of infectious diseases by inhibiting bacterial QS [12]. Therefore, our next aim was to detect the changes in AI-2 biosynthesis. Here we detected significantly low AI-2 induced luminescence response in the sinefungin treated samples that indicates that sinefungin interferes in AI-2 biosynthesis. Similarly, Stroeher et al. 2003 [40] reported the significantly reduced ability of* luxS* mutant strain to elicit bioluminescence in a* V*.* harveyi *AI-2 reporter strain.

Next, we detected changes in gene expression of* luxS* and* pfs* genes. The significantly decreased expressions of* luxS* and* pfs *genes could be due to inhibition of SAM-dependent methyltransferase activity. The encoded Pfs and LuxS enzymes are necessary for biofilm formation and production of AI-2 [14, 41]. In* S. pneumoniae, luxS* is a virulent gene and a central regulator of competence, fratricide, and biofilm formation, and a* luxS* mutant strain displays a significant decrease in pneumococcal biofilm formation [13, 14, 42]. The downregulation of* luxS* and* pfs *genes may decrease the biosynthesis of AI-2 in sinefungin treated samples [40]. In* S. pneumoniae*, AI-1 is absent and AI-2 is the only QS molecule involved in expression of the enzymes for biofilm formation, exotoxin synthesis, and antibiotic resistance factors [19]. Thus, the downexpression of* luxS* and* pfs *genes leads to decreased biosynthesis of AI-2, which in turn disrupts spatial arrangement and build-up of the organized biofilms. Previous studies reported that cultures of* Vibrio cholerae *and enterohemorrhagic* E. coli *(EHEC) strain O157:H7 treated with MTA/SAH nucleosidase inhibitors did not synthesize AI-2 and showed reduced biofilm formation [39]. In a previous study, 5-azacytidine, a hypomethylating drug, demonstrated similar downregulation of genes of AMC [34].

The other reason for inhibitory effect of sinefungin may be accumulation of toxic byproducts of AMC. Recent reports have suggested that substrates of AMC, such as MTA and SAH, are toxic to cells and that bacteria possess MTA/SAH nucleosidase and/or a combination of SAH hydrolase and MTA phosphorylase to remove these inhibitory nucleosides [21]. In* S. pneumoniae*, methionine is converted to SAM by SAM synthetase (encoded by* metK*) and the SAM-dependent methyltransferase produces SAH and methylated products. The detoxification of SAH is carried out by MTA/SAH nucleosidase (Pfs) to produce S-ribosylhomocysteine (SRH) [17, 19]. Then the enzyme S-ribosylhomocysteine lyase (LuxS) further cleaves SRH to homocysteine and 4,5-dihydroxy-2,3-pentanedione, the precursor of the AI-2 QS molecule (Figure 7). Sinefungin is the analog of SAM, in which the S–CH_3_ (sulfonium moiety) of SAM is replaced by a C–NH_3_ (amine). Therefore, sinefungin inhibits SAM-dependent nucleic acid methyltransferases by competing with SAM for occupancy of the methyl donor site on the enzyme [27, 43, 44]. Thus, the presence of sinefungin may interfere with the methylation process and downregulate* luxS* and* pfs* genes, resulting in an imbalance of SAH, SRH, and MTA. Similarly the growth defects were observed in the* Neisseria meningitidis pfs *and* luxS* mutants either due to the accumulation of toxic SAH and MTA or due to metabolic imbalances within the bacteria [45].

The decarboxylated SAM is involved in biosynthesis of spermidine, a polyamine involved in biofilms. Spermidine synthase encoded by* speE* catalyzes the production of spermidine from putrescine and decarboxylated SAM [46]. Previous studies reported* speE* a virulence gene involved in pneumococcal colonization, pneumonia, and invasive infections [47]. The low expression of* speE* gene in biofilms grown with sinefungin indicates that sinefungin also interfere in spermidine biosynthesis.

Only a few molecules have proven effective against pneumococcal biofilms. Domenech et al. reported 80% reduction of pneumococcal biofilms growth with the amidase LytA, and, recently, Sumitomo et al. reported that S-carboxymethylcysteine inhibits pneumococcal adhesion to human alveolar epithelial cells [48, 49]. Similarly, Trappetti et al. showed that neuraminidase inhibitors DANA (i.e., 2,3-didehydro-2-deoxy-N-acetylneuraminic acid), zanamivir, and oseltamivir inhibit the capacity of pneumococci to form sialic acid-dependent biofilms [50]. Here, we first time showed that sinefungin significantly inhibits* in vitro* biofilm growth and* in vivo* middle ear colonization of pneumococci.

In conclusion, sinefungin decreases* in vitro *pneumococci biofilm growth, decreases formation of organised biofilms, decreases AI-2 production, and downregulates expressions of* luxS*,* pfs*, and* speE* genes. Sinefungin also decreases* in vivo* colonization of pneumococci in the middle ear. Therefore, the S-adenosylmethionine (SAM) inhibitors can be used as lead compound for the development of novel antibiofilm agents against pneumococci.

## Figures and Tables

**Table 1:** Primers used for gene expression study.

Genes	Primer sequences	Amplicon size (base pair)
*speE *	F-5′-GACTTTGCTGCAGGGCTAGA-3′ R-5′-AAATGGATCTGTCGCATCGT-3′	120

*luxS *	F-5′-TATGTTCGCTTGATTGGG-3′ R-5′-GCCGGCAGTAGGGATAGAGT-3′	105

*pfs *	F-5′-TTGCTGCTATGCCAGAAGAA-3′ R-5′-TTCCCCAAAACAACTTGCTC-3′	76

*gyrB *	5′-CAGATCAAGAAATCAAACTCCAA-3′ 5′-CAGCATCATCTACAGAAACTC-3′	172

## References

[1] Ispahani P, Slack RC, Donald FE, Weston VC, Rutter N (2004). Twenty year surveillance of invasive pneumococcal disease in Nottingham: serogroups responsible and implications for immunisation. *Archives of Disease in Childhood*.

[2] Kadioglu A, Weiser JN, Paton JC, Andrew PW (2008). The role of *Streptococcus pneumoniae* virulence factors in host respiratory colonization and disease. *Nature Reviews Microbiology*.

[3] Hall-Stoodley L, Hu FZ, Gieseke A (2006). Direct detection of bacterial biofilms on the middle-ear mucosa of children with chronic otitis media. *Journal of the American Medical Association*.

[4] Costerton JW, Stewart PS, Greenberg EP (1999). Bacterial biofilms: a common cause of persistent infections. *Science*.

[5] Wolcott RD, Ehrlich GD (2008). Biofilms and chronic infections. *Journal of the American Medical Association*.

[6] Kania RE, Lamers GE, Vonk MJ (2008). Characterization of mucosal biofilms on human adenoid tissues. *The Laryngoscope*.

[7] Vlastarakos PV, Nikolopoulos TP, Maragoudakis P, Tzagaroulakis A, Ferekidis E (2007). Biofilms in ear, nose, and throat infections: how important are they?. *The Laryngoscope*.

[8] del Prado G, Ruiz V, Naves P, Rodríguez-Cerrato V, Soriano F, del Carmen Ponte M (2010). Biofilm formation by *Streptococcus pneumoniae* strains and effects of human serum albumin, ibuprofen, N-acetyl-l-cysteine, amoxicillin, erythromycin, and levofloxacin. *Diagnostic Microbiology and Infectious Disease*.

[9] García-Castillo M, Morosini MI, Valverde A (2007). Differences in biofilm development and antibiotic susceptibility among *Streptococcus pneumoniae* isolates from cystic fibrosis samples and blood cultures. *Journal of Antimicrobial Chemotherapy*.

[10] Marks LR, Parameswaran GI, Hakansson AP (2012). Pneumococcal interactions with epithelial cells are crucial for optimal biofilm formation and colonization in vitro and in vivo. *Infection and Immunity*.

[11] Bala A, Kumar R, Harjai K (2011). Inhibition of quorum sensing in *Pseudomonas aeruginosa* by azithromycin and its effectiveness in urinary tract infections. *Journal of Medical Microbiology*.

[12] Martin CA, Hoven AD, Cook AM (2008). Therapeutic frontiers: preventing and treating infectious diseases by inhibiting bacterial quorum sensing. *European Journal of Clinical Microbiology and Infectious Diseases*.

[13] Trappetti C, Gualdi L, di Meola L (2011). The impact of the competence quorum sensing system on *Streptococcus pneumoniae* biofilms varies depending on the experimental model. *BMC Microbiology*.

[14] Vidal JE, Ludewick HP, Kunkel RM, Zähner D, Klugman KP (2011). The luxs-dependent quorum-sensing system regulates early biofilm formation by *Streptococcus pneumoniae* strain D39. *Infection and Immunity*.

[15] Beeston AL, Surette MG (2002). *pfs*-dependent regulation of autoinducer 2 production in *Salmonella enterica* serovar Typhimurium. *Journal of Bacteriology*.

[16] Bassler BL, Greenberg EP, Stevens AM (1997). Cross-species induction of luminescence in the quorum-sensing bacterium *Vibrio harveyi*. *Journal of Bacteriology*.

[17] Markham GD, Pajares MA (2009). Structure-function relationships in methionine adenosyltransferases. *Cellular and Molecular Life Sciences*.

[18] Sun J, Daniel R, Wagner-Döbler I, Zeng AP (2004). Is autoinducer-2 a universal signal for interspecies communication: a comparative genomic and phylogenetic analysis of the synthesis and signal transduction pathways. *BMC Evolutionary Biology*.

[19] Winzer K, Hardie KR, Burgess N (2002). LuxS: its role in central metabolism and the in vitro synthesis of 4-hydroxy-5-methyl-3(2H)-furanone. *Microbiology*.

[20] Borchardt RT (1980). S-adenosyl-L-methionine-dependent macromolecule methyltransferases: potential targets for the design of chemotherapeutic agents. *Journal of Medicinal Chemistry*.

[21] Parveen N, Cornell KA (2011). Methylthioadenosine/S-adenosylhomocysteine nucleosidase, a critical enzyme for bacterial metabolism. *Molecular Microbiology*.

[22] Barbes C, Sanchez J, Yebra MJ, Robert-Gero M, Hardisson C (1990). Effects of sinefungin and S-adenosylhomocysteine on DNA and protein methyltransferases from *Streptomyces* and other bacteria. *FEMS Microbiology Letters*.

[23] Fuller RW, Nagarajan R (1978). Inhibition of methyltransferases by some new analogs of S-adenosylhomocysteine. *Biochemical Pharmacology*.

[24] McCammon MT, Parks LW (1981). Inhibition of sterol transmethylation by S-adenosylhomocysteine analogs. *Journal of Bacteriology*.

[25] Paolantonacci P, Lawrence F, Robert-Gero M (1985). Differential effect of sinefungin and its analogs on the multiplication of three *Leishmania* species. *Antimicrobial Agents and Chemotherapy*.

[26] Perez-Leal O, Moncada C, Clarkson AB, Merali S (2011). *Pneumocystis* S-adenosylmethionine transport: a potential drug target. *American Journal of Respiratory Cell and Molecular Biology*.

[27] Zheng S, Hausmann S, Liu Q (2006). Mutational analysis of *Encephalitozoon cuniculi* mRNA cap (guanine-N7) methyltransferase, structure of the enzyme bound to sinefungin, and evidence that cap methyltransferase is the target of sinefungin’s antifungal activity. *The Journal of Biological Chemistry*.

[28] Parsek MR, Val DL, Hanzelka BL, Cronan JE, Greenberg EP (1999). Acyl homoserine-lactone quorum-sensing signal generation. *Proceedings of the National Academy of Sciences of the United States of America*.

[29] Miller ST, Xavier KB, Campagna SR (2004). *Salmonella typhimurium* recognizes a chemically distinct form of the bacterial quorum-sensing signal AI-2. *Molecular Cell*.

[30] Holzman TF, Riley PL, Baldwin TO (1980). Inactivation of luciferase from the luminous marine bacterium *Beneckea harveyi* by proteases: evidence for a protease labile region and properties of the protein following inactivation. *Archives of Biochemistry and Biophysics*.

[31] Oggioni MR, Iannelli F, Ricci S (2004). Antibacterial activity of a competence-stimulating peptide in experimental sepsis caused by *Streptococcus pneumoniae*. *Antimicrobial Agents and Chemotherapy*.

[32] Moscoso M, García E, López R (2006). Biofilm formation by *Streptococcus pneumoniae*: role of choline, extracellular DNA, and capsular polysaccharide in microbial accretion. *Journal of Bacteriology*.

[33] Yadav MK, Chae S, Song J (2012). In vitro *Streptococcus pneumoniae* biofilm formation and in vivo middle ear mucosal biofilm in a rat model of acute otitis induced by S. pneumoniae. *Clinical and Experimental Otorhinolaryngology*.

[34] Yadav MK, Chae SW, Song JJ (2012). Effect of 5-azacytidine on in vitro biofilm formation of *Streptococcus pneumoniae*. *Microbial Pathogenesis*.

[35] Livak KJ, Schmittgen TD (2001). Analysis of relative gene expression data using real-time quantitative PCR and the 2^-ΔΔCT^ method. *Methods*.

[36] Lizcano A, Chin T, Sauer K, Tuomanen EI, Orihuela CJ (2010). Early biofilm formation on microtiter plates is not correlated with the invasive disease potential of *Streptococcus pneumoniae*. *Microbial Pathogenesis*.

[37] Vidal JE, Howery KE, Ludewick HP, Nava P, Klugman KP (2013). Quorum-sensing systems LuxS/Autoinducer 2 and com regulate *Streptococcus pneumoniae* biofilms in a bioreactor with living cultures of human respiratory cells. *Infection and Immunity*.

[38] Zingg JM, Shen JC, Yang AS, Rapoport H, Jones PA (1996). Methylation inhibitors can increase the rate of cytosine deamination by (cytosine-5)-DNA methyltransferase. *Nucleic Acids Research*.

[39] Gutierrez J, Crowder T, Rinaldo-Matthis A, Ho MC, Almo SC, Schramm VL (2009). Transition state analogs of 5′-methylthioadenosine nucleosidase disrupt quorum sensing. *Nature Chemical Biology*.

[40] Stroeher UH, Paton AW, Ogunniyi AD, Paton JC (2003). Mutation of luxS of *Streptococcus pneumoniae* affects virulence in a mouse model. *Infection and Immunity*.

[41] Schauder S, Shokat K, Surette MG, Bassler BL (2001). The LuxS family of bacterial autoinducers: biosynthesis of a novel quorum-sensing signal molecule. *Molecular Microbiology*.

[42] Romao S, Memmi G, Oggioni MR, Trombe MC (2006). LuxS impacts on LytA-dependent autolysis and on competence in *Streptococcus pneumoniae*. *Microbiology*.

[43] Horton JR, Liebert K, Hattman S, Jeltsch A, Cheng X (2005). Transition from nonspecific to specific DNA interactions along the substrate-recognition pathway of dam methyltransferase. *Cell*.

[44] Schluckebier G, Kozak M, Bleimling N, Weinhold E, Saenger W (1997). Differential binding of S-adenosylmethionine S-adenosylhomocysteine and sinefungin to the adenine-specific DNA methyltransferase M. TaqI. *Journal of Molecular Biology*.

[45] Heurlier K, Vendeville A, Halliday N (2009). Growth deficiencies of *Neisseria meningitidis pfs* and *luxS* mutants are not due to inactivation of quorum sensing. *Journal of Bacteriology*.

[46] Shah P, Swiatlo E (2008). A multifaceted role for polyamines in bacterial pathogens. *Molecular Microbiology*.

[47] Shah P, Nanduri B, Swiatlo E, Ma Y, Pendarvis K (2011). Polyamine biosynthesis and transport mechanisms are crucial for fitness and pathogenesis of *Streptococcus pneumoniae*. *Microbiology*.

[48] Domenech M, Garciá E, Moscoso M (2011). In vitro destruction of *Streptococcus pneumoniae* biofilms with bacterial and phage peptidoglycan hydrolases. *Antimicrobial Agents and Chemotherapy*.

[49] Sumitomo T, Nakata M, Yamaguchi M, Terao Y, Kawabata S (2012). *S*-carboxymethylcysteine inhibits adherence of *Streptococcus pneumoniae* to human alveolar epithelial cells. *Journal of Medical Microbiology*.

[50] Trappetti C, Kadioglu A, Carter M (2009). Sialic acid: a preventable signal for pneumococcal biofilm formation, colonization, and invasion of the host. *Journal of Infectious Diseases*.

